# Combined Phacoemulsification and Ex-PRESS Implant with Everting Suture in Primary Angle-Closure Glaucoma: Survival Analysis and Predictive Factors

**DOI:** 10.3390/jcm10040774

**Published:** 2021-02-15

**Authors:** Giuseppe Covello, Pasquale Loiudice, Maria Novella Maglionico, Marco Nardi, Michele Figus, Chiara Posarelli

**Affiliations:** Ophthalmology, Department of Surgical, Medical, Molecular Pathology and Critical Care Medicine, University of Pisa, 56126 Pisa, Italy; giucovello@gmail.com (G.C.); ldcpasquale@gmail.com (P.L.); m.novella.maglionico@gmail.com (M.N.M.); marco.nardi@med.unipi.it (M.N.); michele.figus@unipi.it (M.F.)

**Keywords:** primary angle-closure glaucoma, Ex-PRESS minishunt, everting suture, combined glaucoma surgery, filtering surgery

## Abstract

The purpose of this study was to evaluate the efficacy and safety of combined phacoemulsification and Ex-PRESS implant with everting suture in primary angle-closure glaucoma (PACG) and to examine predictive factors of failure. Twenty-three eyes of 18 patients were enrolled. Data about time of removal of releasable sutures and traction of the everting suture and about changes in intraocular pressure (IOP) were collected, as well as comprehensive ophthalmic examinations. Success was defined by the following criteria: IOP ≤ 18 mmHg (criterion 1); IOP ≤ 15 mmHg (criterion 2); and IOP ≤ 12 mmHg (criterion 3). Success was categorized as complete or qualified, depending on whether it was reached without or with drugs, respectively. Success rate was assessed with Kaplan–Meier survival analysis with a Cox proportional hazard model to adjust for potential confounders. The lowering of IOP and the reduction of medications were statistically significant at every follow-up visit compared with the baseline (*p* < 0.05). The complete success rates were 87%, 70%, and 17% accordingly to criterion 1, 2, and 3; the qualified success rates were 93%, 70%, and 20%, respectively. Most of the complications resolved spontaneously and conservatively. In conclusion, combined phacoemulsification and Ex-PRESS Minishunt implant with everting suture is a safe and effective surgery, even in PACG, lowering IOP and number of medications.

## 1. Introduction

Primary angle-closure glaucoma (PACG) is a leading cause of irreversible blindness, affecting 20.17 million people worldwide, 76.7% of whom are of Asian ethnicity [1]. The number of people with PACG is expected to increase to 23.36 million in 2020 and 32.04 million in 2040, globally [2]. Traditional treatment of PACG relies on laser peripheral iridotomy (LPI) and glaucoma medications with the aim of controlling intraocular pressure (IOP) [3]. In 2016, Azuara-Blanco A. and co-workers [4] demonstrated that clear-lens extraction in new PACG patients showed greater efficacy and was more cost-effective than laser peripheral iridotomy, and that it should be considered as an option for first-line treatment. The Eagle study [4] has highlighted that early clear-lens extraction in PACG could obtain good visual outcomes, a lower IOP, and a delay in surgery.

When previous strategies fail to reach the target pressure and perimetric parameters worsen, surgery is required. The actual gold standard procedure for PACG treatment is trabeculectomy, eventually combined with phacoemulsification [5,6].

However, trabeculectomy is associated with high-risk complications such as hypotony, shallow anterior chamber, malignant glaucoma, and bleb leakage [7]. Ex-PRESS Minishunt (Alcon, Fort Worth, TX, USA) is a stainless-steel glaucoma device that is implanted at the limbus to drain aqueous humor from the anterior chamber to the subconjunctival space, creating a conjunctival bleb, which is like a trabeculectomy [8,9,10,11]. Ex-PRESS Minishunt could be considered as an alternative surgical procedure to trabeculectomy in lowering IOP [12,13,14,15,16,17,18]. Moreover, fewer complications, such as hyphema and encapsulated bleb, have been reported with this procedure [19]. In order to further reduce the aforementioned complications, releasable sutures have been used [20]. Manipulation of the suture tension in the early postoperative days may have considerable advantages—decreasing complication rates and improving success rates—hence slowing the progression of glaucoma [20,21]. Additionally, an everting suture can be applied to lift the scleral flap, avoiding conjunctival invasive manipulation [22].

A deep anterior chamber is essential for Ex-PRESS implantation. Indeed, Ex-PRESS implant is indicated for primary open-angle glaucoma [23]. In recent years, some papers have been published about the efficacy and safety of Ex-PRESS Minishunt implant combined with phacoemulsification in PACG [24,25,26]. In this study, we evaluated the efficacy and safety of combined Ex-PRESS Minishunt and phacoemulsification with everting suture in PACG patients, as well as analyzed potential predictive factors for failure.

## 2. Materials and Methods

We performed a 2-year, retrospective case study of PACG patients who underwent combined Ex-PRESS Minishunt implant and phacoemulsification between January 2016 and December 2017. The study was conducted according to the guidelines of the Declaration of Helsinki and approved by the Area Vasta Nord Ovest Ethical Committee (CEAVNO) with code number 18434_FIGUS. All surgeries were performed by the same surgeon (M.N.) at the Ophthalmology Unit of the Department of Surgical, Medical and Molecular Pathology and Critical Care Medicine, University of Pisa. Inclusion criteria comprised previous diagnosis of PACG (defined by an occludable anterior chamber angle in which the posterior trabecular meshwork was visible for <90° of the angle circumference); elevated IOP (>21 mmHg); a glaucomatous optic disc with a progressive visual field defect despite maximum medical therapy; surgical treatment consisting of combined cataract extraction with Ex-PRESS device implantation; and a postoperative follow-up period of at least two years. Exclusion criteria included uveitis, concurrent retinal or optic neuropathy, and a follow-up period <24 months. We collected data about best-corrected visual acuity (BCVA) expressed as a Logarithm of the Minimum Angle of Resolution (LogMAR), IOP, slit-lamp biomicroscopy, number of medications, and complications at baseline, 1 day after surgery as well as 1, 3, 6, 12, and 24 months after surgery. Moreover, during the first 60 days, patients were visited every week and manipulation of releasable and everting sutures was performed as needed.

### 2.1. Surgical Procedure Description

**Figure 1:**
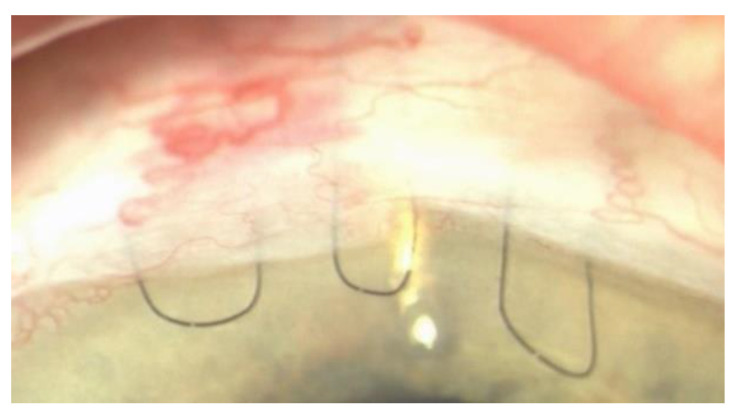
Picture of the Ex-PRESS Minishunt with the two releasable sutures on the sides and the everting suture in the middle.

Combined phacoemulsification and Ex-PRESS Minishunt surgery was performed under retrobulbar anesthesia as follows: a lid speculum was inserted and a 7/0 vycril corneal traction suture was placed. A fornix-based conjunctival flap was sculpted, and a 4 × 4 mm rectangular flap was created. Cellulose sponges soaked in 0.02 mg/mL mitomycin-C were applied under the conjunctival, Tenon’s capsule, and scleral flap for 2 min and 30 s. Irrigation with balanced salt solution was used to wash out residual mitomycin-C solution. Phacoemulsification and subsequent intraocular lens implantation were performed. A single 10-0 nylon stitch was used to close the two corneal incisions for cataract surgery. The scleral flap was lifted, a 25-gauge needle was inserted into the anterior chamber, and the shunt was then placed in the anterior chamber through the ostium created with the needle. The scleral flap was sutured with two releasable 10-0 nylon monofilaments with an everting 10-0 nylon suture [11]. The everting suture was passed through the distal margin of the flap, then through the limbus, and again through the limbus, and finally knotted, making a closed ellipse with a loop on the cornea [22] (Figure 1).

The conjunctiva was closed with three 8-0 silk sutures at the sides of the scleral flap after the conjunctiva and the sclera were sutured together with a single continuous 10-0 nylon suture.

Postoperatively, patients were treated with local chloramphenicol (0.5%)–dexamethasone (0.2%) six times a day for the first two weeks and subsequently with dexamethasone drops tapered along a six-month period.

### 2.2. Success Criteria

Success was defined by the following criteria: IOP ≤ 18 mmHg (criterion 1); IOP ≤ 15 mmHg (criterion 2); and IOP ≤ 12 mmHg (criterion 3). The success was categorized as complete or qualified whether reached without or with drugs, respectively. Failure was considered when IOP was > 21 mmHg on two consecutive follow-up visits 3 months after surgery, with an IOP ≤ 5 mmHg on two consecutive follow-up visits after 3 months in the case of reoperation for glaucoma or loss of light perception vision. Needling revision was not regarded as failure.

### 2.3. Statistical Analysis

Statistical analysis was performed using the SPSS statistical package (version 25.0, IBM, Armonk, NY, USA). Normal distribution of variables was assessed using Kolmogorov–Smirnov and Shapiro–Wilk tests. Analysis of variance (ANOVA) for repeated measures and the Friedman test were used to compare differences over time. A paired t-test and the Wilcoxon signed-rank test were used to compare continuous variables. We assessed the success rate with Kaplan–Meier survival analysis and we conducted a Cox proportional hazard model to adjust for potential confounders including age, sex, preoperative IOP value, and baseline number of glaucoma medications, as well as IOP at 1 day, and then 1, 3, 6, and 12 months postoperative. Other potential confounders were IOP value after first and second releasable suture removal and after everting suture traction; IOP change after releasable suture removal and everting suture traction; and, additionally, time of removal, presence of iridotomy, and occurrence of postoperative complications. Variables with *p* < 0.2 in the univariate model were included in multivariate analysis. A *p* value < 0.05 was considered significant. Moreover, we performed multicollinearity within medication-number-related variables and IOP-related variables. Collinearity diagnostics were obtained, performing correlation analysis between the variables of interest, and then calculating Tolerance and Variable Inflation Factors (VIFs).

## 3. Results

We collected data from about 23 eyes of 18 patients who underwent phacoemulsification and Ex-PRESS Minishunt for PACG. Baseline characteristics are summarized in Table 1.

**Figure 2:**
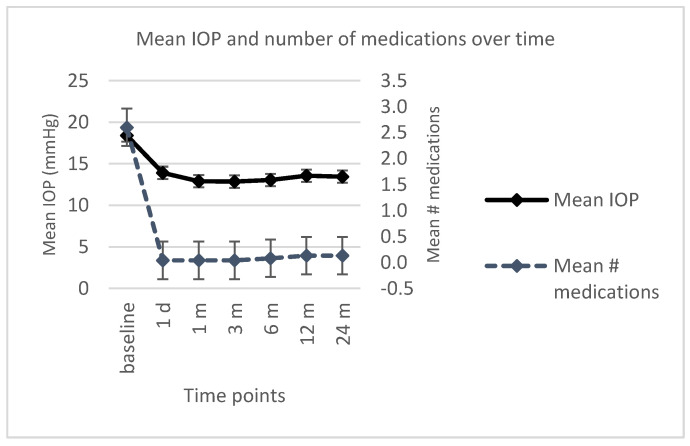
Mean intraocular pressure (IOP) and number of medications at every time point. IOP: intraocular pressure; #: number; d: day; m: months.

The trend of IOP and number of medications over time is displayed in Figure 2. 

The lowering of IOP and the reduction of medications were statistically significant at every follow-up visit compared with the baseline (*p* < 0.05) (Table 2). Analysis of variance for repeated measures showed a significant difference in pairwise comparisons between preoperative IOP and each postoperative follow-up measurement (1 day, and then 1, 3, 6, 12, and 24 months after surgery) (all *p* < 0.05).

Mean IOP values significantly decreased from 17.8 ± 4.3 mmHg to 12.8 ± 3.8 mmHg (*p* < 0.001), from 18.2 ± 4.9 mmHg to 12.5 ± 2.6 mmHg (*p* = 0.015), and from 15.6 ± 0.9 mmHg to 13.1 ± 1.9 mmHg (*p* < 0.001) after the removal of the first and the second releasable suture, and after the everting suture traction, respectively. The mean time for sutures removal was 12.6 ± 6.0 days (median 13, interquartile range (IQR) 8) for the first releasable suture, 22.3 ± 10.3 days (median 20, IQR 15) for the second releasable suture, and 36 ± 6.5 days (median 36, IQR 4) for the everting suture traction. The mean BCVA improved during the follow-up visits from an average value of 0.33 ± 0.01 logMAR preoperatively to a mean value of 0.13 ± 0.19 logMAR at last follow-up visit (Table 2). This improvement was significant at all time points (*p* < 0.05), except for the first postoperative day.

**Figure 3:**
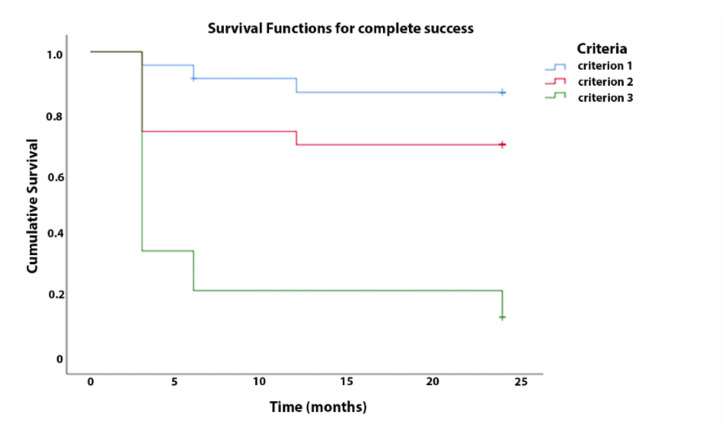
Kaplan–Meier survival analysis for complete success.

The cumulative probability of success at 2 years was 86%, 69%, and 13% accordingly to criterion 1, 2, and 3, respectively, for complete success, and 91%, 69%, and 13%, respectively, for qualified success (Figure 3).

**Figure 4:**
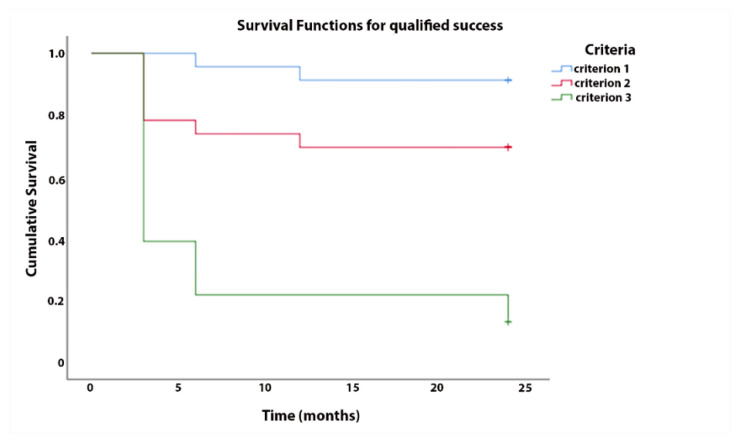
Kaplan–Meier survival analysis for qualified success.

The complete success rates for criterion 1, criterion 2, and criterion 3 were 87%, 70%, and 17%, respectively, and the qualified success rates were 93%, 70%, and 20%, respectively (Figure 4).

Potential risk factors for failure identified with univariate analysis (*p* < 0.2) are displayed in Table 3 and Table 4 for complete and qualified success. Univariate analysis highlighted some potential risks for failure, such as baseline number of medications, IOP at months 6 and 12, time of removal of releasable sutures, or of the everting suture’s traction. Nevertheless, in the multivariate Cox proportional hazards model, none of the covariables were significantly associated with failure either for the complete or qualified success criterion (Supplementary Tables S1 and S2).

No major complications occurred, such as Ex-PRESS protrusion, but five patients experienced minor postoperative complications. Particularly, three patients developed a transient hypotony but only one developed a transient hyphema; both complications appeared in the first day after surgery and resolved spontaneously in a few days. One eye showed a shallow anterior chamber that was treated with atropine 1% drops (twice a day for 7 days). Through managing the wound healing process with releasable sutures removal and everting suture traction, only two patients required a needling procedure with mitomycin-C: one of them at two months after surgery, and the other one at six months after the procedure; no 5-fluorouracil was used.

## 4. Discussion

The aim of this paper was to analyze the efficacy and safety of combined phacoemulsification and Ex-PRESS implant with everting suture in PACG patients and to examine the predictive factors of failure. In recent years, the application of Ex-PRESS Minishunt for PACG has increased [6,7,19,25]. Due to a shallower anterior chamber, Ex-PRESS implant alone is not indicated for PACG because of a higher risk of failure and for a possible contact of the device with the cornea or the iris [27]. This contact could lead to a reduction in corneal endothelium cells or to an iris wound. Moreover, an iris could occlude the shunt. The combination of phacoemulsification and intraocular lens implantation increases the anterior chamber depth and allows for a safer Ex-PRESS implantation [28]. The lens plays an active role in determining anterior chamber depth (ACD) and trabecular-iris angle width. During life, lens volume increases, modifying these two parameters, with a weakening of the ciliary zonules and an anterior displacement of the lens–iris diaphragm [29]. After cataract removal, was observed an increase in anterior chamber depth especially in eyes with the smallest baseline ACD and with a higher central lens thickness [29]. In this retrospective study, a total of 23 eyes with PACG were included. Twelve eyes had previously undergone laser peripheral iridotomy, the first surgical approach for these patients. Despite iridotomy and medical treatment, the visual field’s damage progression was observed. For these patients, the best surgical option turned out to be a combined procedure, due to the role of cataracts in reducing the depth of the anterior chamber and visual acuity. Our results show a significant lowering of IOP and a reduction in the number of medications in all the follow-up visits and a significant increase in visual acuity, with a low complications rate. Our study confirmed the promising results of earlier studies [24,25,26]. Liu and coworkers [25] showed a significant decrease in IOP and number of medications in patients with PACG and cataract who underwent combined phacoemulsification and Ex-PRESS implant with tight sutures of the scleral flap. Particularly, from 20.4 ± 5.4 mmHg preoperatively, they reported an average IOP value of 14.0 ± 3.6 mmHg at last follow-up visit (12 months). The mean number of medications decreased from 3.1 ± 1.2 to 0.3 ± 0.6 at 12 months. These results are similar to those published by Nie and coworkers [26]. In this prospective study, patients with PACG and cataract were followed for up to 36 months. They specified that the scleral flap was sutured using two permanent sutures and one or two releasable 10-0 nylon sutures, although IOP values before and after Argon laser suture lysis were not reported. The mean preoperative IOP was 28.43 ± 12.93 mmHg and decreased to 15.35 ± 4.02 mmHg 3 years after surgery. The number of medications decreased from 2.47 ± 1.89 at baseline to 0.28 ± 0.76 3 years postoperatively [26]. Lan and coworkers [24] published data about 60 patients. In this comparative study, 30 patients with primary open-angle glaucoma and 30 with PACG underwent combined phacoemulsification and Ex-PRESS implant with tight sutures of the scleral flap. They observed a significant reduction from baseline to the last follow-up visit (36 months) in both groups, even if the primary open-angle glaucoma group seemed to have lower postoperative IOP but a higher risk of hypotony [24].

One of the principal limitations of the Ex-PRESS Minishunt is the long-term efficacy. It is known that the main challenge of this surgical approach is maintaining bleb filtration over the years [30]. Some changes have been made to preserve efficacy and ensure safety [30,31], such as releasable sutures and the use of antimetabolites. The withdrawal of the suture tension in the early postoperative period may decrease complication rates and improve success rates [20]. However, this maneuver may not be enough to reopen a pathway for aqueous outflow and to lower IOP [22]. An everting suture could be applied as an adjunctive strategy to control the aqueous drainage. It is known that the wound-healing process of the scleral flap starts a few minutes after surgery and continues for several months if antimetabolites have been used. A dynamic process of remodeling leads to a final, mature scar. It is crucial to observe and monitor bleb formation every week to determine the degree of scar formation. Hence, placing and removing the scleral flap sutures allows us to control postoperative IOP by titrating the flow of aqueous through the scleral flap valve [22]. If releasable sutures are inadequate, everting suture may lift the flap. Figus and coworkers stated that the best time to pull on the everting suture is probably when scarring surrounds the flap, between the third and the sixth weeks after surgery, though individual factors could influence the healing process and modify this timeline [22].

Our results highlight the efficacy of this surgical strategy and the importance of releasable and everting sutures in order to avoid early complications. Particularly, only three eyes developed a transient hypotony that resolved spontaneously. The complete success rates for criterion 1, criterion 2, and criterion 3 were 87%, 70%, and 17%, respectively, and the qualified success rates were 93%, 70%, and 20%, respectively. These results are in accordance with those reported in previous studies [24,25,26]. Moreover, in a multivariate Cox proportional hazards model, no predictive risk factors for failure were found according to all the criteria for both complete and qualified success. Although univariate analysis has shown some potential risks for failure, such as baseline IOP, time of removal of releasable, or everting suture, in a multivariate model, none of the covariables were significantly associated with failure.

Limitations of this study include its retrospective nature, the small sample size, the relatively short follow-up time, and the absence of a control group. A prospective, randomized controlled clinical trial should be performed to compare combined phacoemulsification with Ex-PRESS Minishunt implantation or trabeculectomy. Perimetric data are essential to evaluate the progression of glaucoma beyond IOP reduction. Furthermore, for a future research purpose, another relevant parameter to be considered in glaucoma implant surgery is corneal endothelial cell count [32]. Nevertheless, Ex-PRESS Minishunt, compared with trabeculectomy and tubes, seems to be a safer procedure regarding the risk of endothelial cell loss [32,33].

## 5. Conclusions

In conclusion, our data confirm the efficacy and safety of combined phacoemulsification and Ex-PRESS Minishunt implantation with everting suture even in PACG, opening new frontiers and increasing surgical options for this challenging type of glaucoma.

## Figures and Tables

**Table 1:** Baseline characteristics.

Parameter	Results
Age	68.7 ± 8.9
Gender	
Male	27 (39.1%)
Female	42 (60.9%)
Laterality	
Right	33 (47.8%)
Left	36 (52.2%)
Baseline IOP (mmHg)	18.4 ± 0.6
Baseline *n*. of medications	2.6 ± 0.1
Baseline BCVA (logMAR)	0.33 ± 0.01
Previous iridotomy	12 (52.2%)

All values are shown as mean ± standard deviation and percentage between brackets. IOP: intraocular pressure; BCVA: best corrected visual acuity.

**Table 2:** Postoperative outcomes at every time point versus baseline values.

	Mean IOP in mmHg	Mean Number of Medications	Mean BCVA
Baseline	18.4 ± 0.6	2.6 ± 0.1	0.33 ± 0.01
1 day	13.9 ± 4.6 *	0.04 ± 0.2 *	0.23 ± 0.17
1 month	12.9 ± 1.87 *	0.04 ± 0.2 *	0.18 ± 0.17 *
3 months	12.86 ± 2.66 *	0.04 ± 0.2 *	0.20 ± 0.21 *
6 months	13.04 ± 2.33 *	0.08 ± 0.28 *	0.17 ± 0.19 *
12 months	13.56 ± 2.38 *	0.13 ± 0.34 *	0.16 ± 0.20 *
24 months	13.45 ± 1.99 *	0.13 ± 0.34 *	0.13 ± 0.19 *

All values are shown as mean ± standard deviation. IOP: intraocular pressure; BCVA: best corrected visual acuity; *: statistically significant value (*p* < 0.05).

**Table 3:** Risk factors for failure: results from univariate Cox regression analysis for complete success.

Risk Factor	Criterion 1			Criterion 2			Criterion 3		
	HR	95% CI	P	HR	95% CI	P	HR	95% CI	P
Age (per decade)	1.073	00926–1.243	0.350	0.971	0.895–1.053	0.471	0.998	0.948–1.051	0.944
As continuous variable	1.556	0.481–5.037	0.460	0.626	0.308–1.274	**0.196**	0.935	0.604–1.448	0.935
Gender	0.291	0.026–3.214	0.314	0.471	0.105–2.105	0.324	0.256	0.2237–1.467	0.256
Baseline IOP	1.123	0.938–1.347	0.207	1.001	1.001–0.860	0.990	0.982	0.899–1.073	0.687
Baseline n. of medications	4.233	0.665–26.937	**0.126**	1.231	0.494–3.064	0.655	1.116	0.662–1.883	0.680
N. of medications at 1 month	1.00	0.00–14926.77	1.00	4.400	0.514–37.661	**0.176**	1.571	0.207–11.950	0.662
N. of medications at 3 months	1.132	0.025–14638.27	0.986	3.540	0.567–32.621	0.396	0.682	0.507–1.684	0.253
N. of medications at 6 months	14.849	0.891–247.51	**0.060**	5.250	0.962–28.663	**0.056**	1.615	0.625–7.158	0.528
N. of medications at 12 months	22.293	1.922–258.62	**0.013**	6.779	1.369–33.582	**0.019**	1.667	0.470–5.906	0.429
IOP at day 1	1.094	0.865–1.383	0.453	0.842	0.674–1.053	**0.132**	1.005	0.891–1.133	0.936
IOP at month 3	1.330	0.752–2.352	0.327	1.191	0.859–1.652	0.293	1.357	1.078–1.708	**0.009**
IOP at month 6	2.713	1.190–6.189	**0.018**	1.186	0.846–1.661	0.322	1.318	1.061–1.636	**0.012**
IOP at month 12	1.843	0.949–3.576	**0.071**	1.396	0.978–1.994	**0.066**	1.315	1.058–1.636	**0.014**
IOP before 1st releasable	1.427	1.034–1.969	**0.031**	0.906	0.736–1.115	0.350	0.998	0.895–1.114	0.976
IOP after 1st releasable	1.283	0.988–1.667	**0.061**	0.906	0.735–1.117	0.356	0.939	0.724–1.071	0.348
IOP change after 1st releasable	1.181	0.855–1.631	0.312	1.009	0.802–1.270	0.938	1.081	0.943–1.240	0.263
IOP before 2nd releasable	17.876	0.034–9528.05	0.368	1.029	0.895–1.184	0.686	1.046	0.965–1.134	0.269
IOP after 2nd releasable	1.223	0.741–2.0149	0.431	0.951	0.716–1.263	0.730	1.066	0.900–1.264	0.458
IOP change after 2nd releasable	1.218	0.997–1.488	**0.054**	1.019	0.890–1.166	0.789	1.021	0.939–1.110	0.630
IOP before everting	2.593	0.929–7.239	**0.069**	0.781	0.350–1.744	0.546	1.236	0.793–1.928	0.349
IOP after everting	1.307	0.835–2.047	0.242	1.238	0.883–1.735	0.215	1.182	0.958–1.457	**0.118**
IOP change after everting	0.909	0.532–1.554	0.727	0.784	0.584–1.052	**0.105**	0.872	0.700–1.087	**0.224**
Time of removal of 1st releasable	1.017	0.850–1.218	0.851	1.013	0.901–1.139	0.827	0.949	0.787–1.026	**0.186**
Time of removal of 2nd releasable	0.991	0.880–1.116	0.880	1.045	0.995–1.098	**0.075**	0.991	0.942–1.04.3	0.733
Time of everting suture removal	0.983	0.835–1.158	0.840	1.025	0.907–1.159	0.690	0.958	0.899–1.021	**0.188**
Iridotomy (yes vs. no)	0.454	0.041–5.013	0.519	1.217	0.272–5.437	0.797	1.289	0.531–3.132	0.575
Complications (yes vs. no)	0.547	0.050–6.041	0.623	30.330	0.016–578.85	0.376	0.949	0.342–2.634	0.921

Significant values are shown in bold. HR: hazard ratio; CI: confidence interval; P: *p*-value; IOP: intraocular pressure; N: numbers.

**Table 4:** Risk factors for failure: results from univariate Cox regression analysis for qualified success.

Risk Factor	Criterion 1			Criterion 2			Criterion 3		
	HR	95% CI	P	HR	95% CI	P	HR	95% CI	P
Age (per decade)	2.414	0.477–12.211	0.287	0.628	0.311–1.267	**0.194**	0.928	0.596–1.443	0.739
As continuous variable	1.115	0.907–1.39	0.301	0.971	0.896–1.053	0.475	0.997	0.954–1.051	0.902
Gender	0.606	0.038–9.702	0.723	0.470	0.105–2.102	0.323	0.615	0.251–1.509	0.288
Baseline IOP	1.072	0.842–1.366	0.571	1.00	0.859–1.164	0.999	0.977	0.894–1.067	0.603
Baseline n. of medications	7.261	0.593–88.850	**0.121**	1.229	0.485–3.114	0.664	1.111	0.656–1.881	0.695
N. of medications at 1 month	0.046	0.00–4.339	0.840	4.202	0.490–36.020	**0.190**	0.946	0.123–7.207	0.957
N. of medications at 3 months	0.677	0.03–4.352	0.760	3.219	0.265–5–897	**0.154**	0.957	0.003–5.297	0.840
N. of medications at 6 months	0.043	0.00–8766.60	0.774	6.656	1.030–31.044	**0.046**	1.229	0.282–5.352	0.783
N. of medications at 12 months	6.498	0.406–103.90	**0.186**	7.745	1.528–39.259	**0.013**	1.386	0.401–4.789	0.606
IOP at day 1	1.062	0.782–1.443	0.700	0.832	0.662–1.045	**0.114**	1.001	0.886–13131	0.983
IOP at month 3	7.378	0.399–136.51	**0.179**	1.214	0.986–1.696	0.256	1.441	1.125–1.845	**0.004**
IOP at month 6	3.952	0.914–17.084	**0.066**	1.204	0.853–1.700	0.291	1.288	1.049–1.583	**0.016**
IOP at month 12	1.983	0.812–4.841	**0.133**	1.435	0.993–2.073	**0.054**	1.292	1.051–1.587	**0.015**
IOP before 1st releasable	1.089	0.825–1.438	0.547	0.894	0.723–1.105	0.298	0.984	0.885–1.093	0.758
IOP after 1st releasable	1.301	0.922–1.837	**0.134**	0.901	0.733–1.108	0.324	0.927	0.810–1.059	0.264
IOP change after 1st releasable	0.755	0.396–1.441	0.394	1.005	0.799–1.263	0.969	1.056	0.930–1.200	0.402
IOP before 2nd releasable	1.120	0.946–1.325	**0.189**	1.027	0.892–1.181	0.715	1.026	0.951–1.106	0.511
IOP after 2nd releasable	1.319	0.666–2.610	0.427	0.949	0.714–1.262	0.720	1.069	0.901–1.269	0.442
IOP change after 2nd releasable	0.920	0.656–1.290	0.629	1.015	0.886–1.163	0.831	1.005	0.929–1.087	0.906
IOP before everting	3.892	1.086–13.946	**0.037**	0.757	0.328–1.745	0.513	1.251	0.800–1.956	0.326
IOP after everting	1.447	0.871–2.402	**0.153**	1.274	0.897–1.809	**0.177**	1.198	0.970–1.479	**0.094**
IOP change after everting	0.872	0.470–1.619	0.665	0.761	0.562–1.031	**0.078**	0.861	0.690–1.075	**0.186**
Time of removal of 1st releasable	1.110	0.930–1.326	0.248	1.014	0.903–1.138	0.819	0.954	0.884–1.029	0.219
Time of removal of 2nd releasable	1.020	0.915–1.137	0.719	1.052	1.00–1.106	**0.051**	0.993	0.944–1.045	0.788
Time of everting suture traction	0.975	0.799–1.190	0.802	1.025	0.905–1.161	0.698	0.955	0.895–1.019	**0.164**
Iridotomy (yes vs. no)	0.013	0.00–1202.91	0.458	1.194	0.267–5.335	0.817	1.254	0.518–3.035	0.616
Complications (yes vs. no)	4.025	0.251–64.627	0.326	0.032	0.00–55.342	0.366	1.077	0.388–2.988	0.887

Significant values are shown in bold. HR: hazard ratio; CI: confidence interval; P: *p*-value; IOP: intraocular pressure; N: numbers.

## Data Availability

The data presented in this study are available on request from the corresponding author. The data are not publicly available due to restrictions from the Ethical Committee (Area Vasta Nord Ovest Ethical Committee, CEAVNO).

## Supplementary Materials

The following are available online at https://www.mdpi.com/2077-0383/10/4/774/s1, Table S1: Risk factors for failure, results from multivariate Cox Regression analysis (complete success), Table S2: Risk factors for failure, results from multivariate Cox Regression analysis (qualified success).
