# Supplementary material for: Combined Phacoemulsification and Ex-PRESS Implant with Everting Suture in Primary Angle-Closure Glaucoma: Survival Analysis and Predictive Factors
Source: J Clin Med. 2021 Feb 15;10(4):774. doi: 10.3390/jcm10040774 (PMC7919368; doi:10.3390/jcm10040774)
Supplement: Supplementary file 1 [file jcm-10-00774-s001.pdf]

Table S1. Risk factors for failure, results from multivariate Cox regression analysis (complete success)

Risk factor	Criterion 1			Criterion 2			Criterion 3		
	HR	95% CI	P	HR	95% CI	P	HR	95% CI	P
As continuous variable	-	-	-	0.975	0.831-1.145	0.761	-	-	-
Baseline n. of medications	1.344	0.00-2.091	0.992	-	-	-	-	-	-
N. of medications at 1 month	-	-	-	9.859	0.01-9342.9	0.513	-	-	-
N. of medications at 6 months	1.049	0.00-1.495	0.999	0.425	0.00-1567.34	0.838	-	-	-
N. of medications at 12 months	6350.76	0.00-1.334	0.932	6.741	0.008-6766.39	0.580	-	-	-
IOP at day 1	-	-	-	0.811	0.477-1.377	0.437	-	-	-
IOP at month 3	-	-	-	-	-	-	1.198	0.863-1.662	0.280
IOP at month 6	2.993	0.00-2146104	0.916	-	-	-	1.078	0.776-1.498	0.653
IOP at month 12	1.013	0.00-5.466	0.999	1.135	0.549-2.346	0.732	1.120	0.781-1.251	0.477
IOP before 1st releasable	1.321	0.005-383.032	0.923	-	-	-	-	-	-
IOP after 1st releasable	1.945	0.005-764.910	0.827	-	-	-	-	-	-
IOP change after 2nd releasable	0.944	0.00-8444.347	0.990	-	-	-	-	-	-
IOP before everting	0.276	0.00-6.039	0.971	-	-	-	-	-	-
IOP after everting	-	-	-	-	-	-	-	-	-
IOP change after everting	-	-	-	1.052	0.239-4.624	0.947	0.988	0.499-2.281	0.922
Time of removal of 1st releasable	-	-	-	-	-	-	0.907	0.795-1.035	0.149
Time of removal of 2nd releasable	-	-	-	1.053	0.829-1.338	0.673	-	-	-
Time of everting removal	-	-	-	-	-	-	1.043	0.925-1.178	0.495

HR: hazard ratio; CI: confidence interval; P: p-value; IOP: intraocular pressure; N: numbers.

Table S2. Risk factors for failure, results from multivariate Cox regression analysis (qualified success)

Risk factor	Criterion 1			Criterion 2			Criterion 3		
	HR	95% CI	P	HR	95% CI	P	HR	95% CI	P
Age (per decade)	-	-	-	0.817	0.186-3.584	0.789	-	-	-
Baseline n. of medications	3.074	0.00-2.374	0.936	-	-	-	-	-	-
N. of medications at 1 month	-	-	-	0.037	0.00-60624.04	0.652	-	-	-
N. of medications at 3 months	-	-	-	-	-	-	-	-	-
N. of medications at 6 months	-	-	-	0.003	0.00-1991030	0.650	-	-	-
N. of medications at 12 months	10.899	0.00-3.701	0.937	83327.769	0.00-5.475	0.373	-	-	-
IOP at day 1	-	-	-	1.044	0.371-2.936	0.935	-	-	-
IOP at month 3	1.586	0.00-1733128	0.948	-	-	-	1.375	0.954-1.982	0.087
IOP at month 6	-	-	-	-	-	-	0.963	0.619-1.498	0.867
IOP at month 12	0.637	0.00-1863.993	0.912	1.727	0.330-9.044	0.517	1.104	0.792-1.539	0.561
IOP after 1st releasable	2.611	0.001-13143.137	0.825	-	-	-	-	-	-
IOP before 2nd releasable	0.748	0.00-1794.444	0.942	-	-	-	-	-	-
IOP before everting	3.191	0.00-8.758	0.925	-	-	-	-	-	-
IOP after everting	2.269	0.00-195050.386	0.888	0.045	0.00-6.675	0.224	1.131	0.563-2.269	0.730
IOP change after everting	-	-	-	0.020	0.00-129.055	0.384	1.121	0.524-2.398	0.768
Time of removal of 2nd releasable	-	-	-	0.912	0.418-1.991	0.817	-	-	-
Time of everting traction	-	-	-	-	-	-	0.992	0.892-1.103	0.884

HR: hazard ratio; CI: confidence interval; P: p-value; IOP: intraocular pressure; N: numbers.
